# Draft genome sequence of an uncultured archaeon from Antarctic endolithic communities

**DOI:** 10.1128/mra.00427-25

**Published:** 2025-07-23

**Authors:** Claudia Coleine, Diego Micheletti, Massimo Pindo, Simone Larger, Erika Stefani, Federico Biagioli, Jason E. Stajich, Claudio Donati

**Affiliations:** 1 Department of Ecological and Biological Sciences, University of Tuscia 19054, Viterbo, Italy; 2 Research and Innovation Centre, Fondazione Edmund Mach 377369 , San Michele all'Adige, Italy; 3 Department of Microbiology and Plant Pathology, Institute of Integrative Genome Biology, University of California, Riverside 117242, Riverside, California, USA; 4 Institute for Integrative Genome Biology, University of California, Riverside, Riverside, California, USA; Portland State University, Portland, Oregon, USA

**Keywords:** Antarctica, extremophiles, archaea, microbes, metagenomics

## Abstract

A draft genome sequence was assembled and annotated for an uncultured archaeon reconstructed from shotgun metagenomes obtained from Antarctic endoliths. The assembled genome is 1.99 megabases and encodes 2,405 predicted protein-coding genes. This genome sequence provides insights into the microbial diversity and functional potential of extremophiles inhabiting Antarctic rock environments.

## ANNOUNCEMENT

Endolithic microbial communities in Antarctica thrive in one of the most extreme environments on Earth (1, 2). These communities inhabit rock airspaces, exploiting microenvironments that allow them to survive at the limits of habitability (3). They host highly adapted microbes that sustain metabolism through trace gas oxidation and atmospheric chemosynthesis under extreme oligotrophic conditions (4–7). Here, we report the draft genome sequence of an uncultured *Nitrosocosmicus* archaeon reconstructed from shotgun metagenomes from Antarctic endolithic communities.

* Nitrosocosmicus* belongs to the phylum Thaumarchaeota, a group of ammonia-oxidizing archaea (8, 9), which is known for the ability to oxidize ammonia under extreme conditions (10, 11). The discovery of *Nitrosocosmicus* in Antarctic endoliths highlights ammonia oxidation as a key survival strategy for Thaumarchaeota in nutrient-poor cold deserts (12). Sequencing this *Nitrosocosmicus* archaeon might reveal insights into microbial diversity, survival, and biogeochemical roles in Antarctic ecosystems (13, 14).

 A quartz was collected on Kay Island (Northern Victoria Land, Antarctica; 74.07°S, 165.31°E) in sterile bags, stored at −20°, and shipped to the University of Tuscia. Environmental DNA was extracted from 1 g of crushed rock using the DNeasy PowerSoil Pro Kit (Qiagen, Germany) (15). DNA quality was assessed by the 4150 TapeStation system and quantified using the Qubit dsDNA HS Assay Kit (Life Technologies, USA) (16). Illumina libraries were constructed using the Kapa Hyperplus Kit (Roche). Full shotgun metagenomic sequencing was performed using the Illumina NovaSeq6000 platform, generating paired-end 2 × 150 bp reads. The total number of reads was 82,718,564 reads. Raw reads were quality trimmed and filtered using BBDuk (BBMap v38.79). During the quality filtering procedure: (i) raw reads were quality trimmed to Q6 using the Phred algorithm; (ii) reads that contained 4 or more “*N*” bases, had an average quality below 10, and shorter than 50 bp or under 50% of the original length were removed. Assembly was performed using SPAdes v3.15.1 (metaSPAdes mode, k-mers: 21, 33, 55, 77, 99) (17). Contigs were binned into metagenome-assembled genomes (MAGs) using MetaBAT2 v2.12.1, with coverage depth estimated via Bowtie2 and Samtools (18, 19). Genomes completeness and contamination were assessed using CheckM v1.1.2 (20). MAGs were classified as high quality (>90% completeness, <5% contamination) or medium quality (≥50% completeness, <10% contamination) (6). A high-quality archaeal MAG was taxonomically classified using the metashot/prok-classify v1.2.1 workflow (https://github.com/metashot/prok-classify, parameters: –gtdbtk_db release202), which integrates the Genome Taxonomy Database Toolkit (GTDB-Tk) v1.5.0 (21).

 The assembled genome of *Nitrosocosmicus* sp. Mars-44.bin.73 is 1.99 Mb in size, with 72.49% completeness and 3.4% contamination. The genome consists of 371 contigs, with an N50 contig length of 6,024 bp and an N50 scaffold length of 6,098 bp. The longest contig and scaffold measure 23,677 bp, while the mean contig and scaffold lengths are 5,363 and 5,606 bp, respectively. The GC content is 34.4% with a coding density of 72.45%. Gene prediction and functional annotation were performed using Prokka v1.14.5 (22), which identified 2,405 predicted protein-coding genes, 35 tRNA genes, and 1 5S rRNA gene. Functional annotation was further refined using EggNOG-mapper v2.1.4 (23), which assigned functions to the predicted genes.

## Data Availability

This Whole Genome Shotgun project has been deposited at DDBJ/ENA/GenBank under the accession JBNJLU000000000. The version described in this paper is version JBNJLU010000000, and it is associated with BioProject PRJNA905198 (Biosample SAMN31867300).
